# Reduced-dose obinutuzumab induces remission in refractory ANCA-associated vasculitis: a report of 16 cases

**DOI:** 10.3389/fimmu.2025.1624234

**Published:** 2025-08-29

**Authors:** Wenyi Wu, Juan Wang, Sheng Chen

**Affiliations:** Department of Rheumatology, Renji Hospital, School of Medicine, Shanghai Jiao Tong University, Shanghai, China

**Keywords:** ANCA-associated vasculitis, obinutuzumab, efficacy, safety, B cells

## Abstract

**Objective:**

Rituximab remains the standard-of-care anti-CD20 therapy for anti-neutrophil cytoplasmic antibody (ANCA)-associated vasculitis (AAV). Obinutuzumab, a next-generation, glycoengineered anti-CD20 monoclonal antibody with enhanced B-cell-depleting capacity, may offer superior efficacy. We evaluated the efficacy and safety of reduced-dose obinutuzumab in 16 patients with active, refractory AAV at a single center in China.

**Methods:**

In this retrospective chart review, we evaluated 16 consecutive patients who received reduced-dose obinutuzumab (most commonly 1,000 mg for induction) after failure to achieve remission with cyclophosphamide (CTX) and/or rituximab (RTX) or who presented with severe, treatment-naïve disease. Primary endpoints were complete remission (CR) rates at 24 and 76 weeks. Secondary endpoints included changes in renal function, inflammatory biomarkers, and immune reconstitution. Adverse events were prospectively recorded.

**Results:**

The median age at obinutuzumab initiation was 44.5 years (IQR 31–54.3); 10 (62.5%) were men. The mean Birmingham Vasculitis Activity Score (BVAS) was 13.5 ± 6.4. There were 12 patients (75%) who had relapsing disease refractory to CTX/RTX, whereas four treatment-naïve patients presented with multiorgan failure. CR was achieved in 8/16 patients (50%) at week 24 and 13/16 patients (81.3%) at week 76. Obinutuzumab induced rapid clinical remission, suppressed systemic inflammation, achieved peripheral B-cell depletion, rendered ANCA-negative, and improved renal and pulmonary outcomes. No severe infections occurred. Seven patients (43.8%) developed treatment-emergent infections, predominantly respiratory (75%).

**Conclusion:**

Reduced-dose obinutuzumab demonstrates sustained remission in refractory or relapsing active AAV, achieving high long-term remission rates with an acceptable safety profile. No severe invasive infections were observed.

## Highlights

• Reduced-dose obinutuzumab induced sustained remission in patients with relapsed or refractory ANCA-associated vasculitis.

## Introduction

Anti-neutrophil cytoplasmic antibody (ANCA)-associated vasculitis (AAV) is a systemic autoimmune condition characterized by necrotizing inflammation of small- to medium-sized blood vessels, frequently resulting in multiorgan involvement. The kidneys are affected in more than 75% of cases, followed by the lungs (50%–70%), the upper respiratory tract, the skin, and, less commonly, the eyes and the peripheral nervous system (1, 2). The clinical course of the disease is typified by rapid progression, cumulative organ damage, and frequent relapses; approximately 40% of patients relapse within 5 years. Renal involvement is particularly ominous, with 20%–40% of patients progressing to end-stage renal disease (ESRD), a complication that substantially worsens the long-term prognosis.

B cells are central to the pathogenesis of AAV. The 2022 EULAR guidelines recommend a combination of glucocorticoids in combination with either rituximab (RTX) or cyclophosphamide (CTX) for life-threatening or organ-threatening AAV (3). However, there is no universally accepted alternative treatment for patients who fail or are intolerant to RTX/CTX, highlighting the need for additional options.

Therapeutic anti-CD20 monoclonal antibodies are classified as Type I or Type II according to their mechanism of action. Obinutuzumab is a humanized, glycoengineered Type II anti-CD20 monoclonal antibody that induces more profound and durable B-cell depletion than Type I agents, such as RTX, via enhanced FcγRIII binding and direct induction of programmed cell death (4–6). Although currently licensed for rituximab-refractory follicular lymphoma, obinutuzumab has demonstrated efficacy in other autoimmune diseases, such as systemic lupus erythematosus and PLA2R-associated membranous nephropathy (7, 8). Preliminary data also indicate renal-protective effects, which are particularly relevant for AAV (9, 10). However, robust evidence for obinutuzumab in AAV is limited. The present study was designed to evaluate obinutuzumab’s ability to induce and maintain remission in patients with AAV refractory to CTX and/or RTX or presenting with severe disease activity.

## Methods

### Study design and patients

This single-center, retrospective chart review enrolled 16 adults with active AAV who either had severe disease activity or were refractory to CTX and/or RTX. Given that ANCA serotype (proteinase 3 [PR3]-ANCA and myeloperoxidase [MPO]-ANCA) is a stronger predictor of clinical presentation, disease course, treatment response, and comorbidities compared with traditional granulomatosis with polyangiitis (GPA) and microscopic polyangiitis (MPA) classification, patients were stratified by ANCA subtype. The majority of patients received a single 1,000-mg exploratory induction dose of obinutuzumab; some were maintained on either RTX or obinutuzumab. All underwent 76 weeks of follow-up in the Rheumatology Department at Shanghai Renji Hospital. Baseline characteristics and treatment details are listed in **Table 1** and **Supplementary Table 1**. The study was approved by the Renji Hospital Ethics Committee (IRB no. LY2025 - 197-A), and all patients provided written informed consent.

**Table 1:** Baseline characteristics of AAV patients who received obinutuzumab.

Characteristics	N=16
Age (years) when started on obinutuzumab, median (IQR)	44.5 (31–54.3)
Age (years) at disease onset, median (IQR)	37 (29.5–53.3)
Duration of the disease (months), median (IQR)	17 (13–20.5)
BVAS, mean (SD)	13.5 (6.42)
Gender	
Male patients	10 (62.5)
Female patients	6 (37.5)
Status	
Newly diagnosed	4 (25)
Relapsing disease	12 (75)
ANCA positivity	
PR3-ANCA+	12 (75)
MPO-ANCA+	4 (25)
System involvement	
Kidney	6 (37.5)
Lung	10 (62.5)
ENT	4 (25)
Nervous	2 (12.5)
Eyes	5 (31.3)
Symptom	
Fever	7 (43.8)
Arthritis	3 (18.8)
Myalgia	3 (18.8)
Oral prednisone at baseline (mg/day)	
15	1 (6.3)
25	1 (6.3)
40	3 (18.8)
50	3 (18.8)
60	8 (50)
Previous therapy	
CTX	10 (62.5)
Mycophenolate mofetil	5 (31.3)
RTX	4 (25)
Methotrexate	6 (37.5)
Azathioprine	4 (25)
Cyclosporin A	1 (6.3)
Leflunomide	1 (6.3)

AAV, anti-neutrophil cytoplasmic antibody (ANCA)-associated vasculitis; IQR, interquartile range; MPO, myeloperoxidase; PR3, proteinase 3; ANCA, anti-neutrophil cytoplasmic antibody; BVAS, Birmingham Vasculitis Activity Score; SD, standard difference; ENT, ear, nose, and throat; CTX, cyclophosphamide; and RTX, rituximab.

All patients met the MPA or GPA criteria, as defined by the American College of Rheumatology/European Alliance of Associations for Rheumatology (11, 12) and the Chapel Hill Consensus Conference (13).

### Outcome definitions

The primary endpoint was the complete remission (CR) rate at 24 weeks and throughout the 76-week follow-up period. CR was defined as a Birmingham Vasculitis Activity Score (BVAS) of zero and an oral prednisone dose of 10 mg/day or less (14). Response was defined as a ≥50% reduction in BVAS with no new manifestations (3). Relapse was defined as the new emergence or recurrence of one or more BVAS items after remission (15).

Secondary outcomes included changes in laboratory parameters and radiographic improvements on chest imaging. Laboratory parameters included inflammatory markers [C-reactive protein level (CRP), erythrocyte sedimentation rate (ESR)] and immunological markers [ANCA titers (quantified using a commercial MPO/PR3-ELISA kit, positive cutoff ≥1 AU), B-cell counts, and serum immunoglobulin levels]. Renal function was monitored using serum creatinine, estimated glomerular filtration rate (eGFR), and change in proteinuria as a surrogate for long-term renal survival (16).

Adverse events were carefully recorded to assess the safety profile of obinutuzumab treatment. These included infections and infusion-related reactions occurring within 24 h post-administration.

### Statistical analysis

Categorical variables are presented as percentages (%), whereas continuous variables are presented as medians with interquartile range (IQR). Statistical significance was determined using a two-sided P-value ≤0.05. All statistical analyses were performed using the R software, version 4.3.2 (R Foundation for Statistical Computing), and GraphPad Prism 8.

## Results

### Patient characteristics

The baseline characteristics of the 16 enrolled patients are presented in **Table 1** and **Supplementary Table 1**. The median age at the first obinutuzumab infusion was 44.5 years (IQR 31–54.3); 10 participants (62.5%) were men and 6 (37.5%) were women. PR3-ANCA was detected in 12 patients (75%), and the mean BVAS was 13.5 ± 6.4. Multiorgan involvement was observed in 13 patients: renal (37.5%), pulmonary (62.5%), and ear–nose–throat (25%). Fever was documented in seven patients (43.8%). There were 12 patients (75%) who received obinutuzumab for relapsing disease, of whom 11 had been previously treated with CTX and/or RTX. Four patients (all PR3-ANCA-positive) received obinutuzumab as first-line therapy immediately after diagnosis; each exhibited multiorgan disease and markedly elevated disease activity. In this subgroup, two developed acute bilateral profound sensorineural hearing loss accompanied by neuroimaging evidence of central nervous system vasculitis, and one presented with fulminant renal failure requiring urgent dialysis.

### Outcomes

#### Primary outcomes

We assessed both short-term (24 weeks) and long-term (76 weeks) outcomes following obinutuzumab administration. The primary outcomes are summarized in **Table 2**. All 16 patients completed the 24-week assessment; one patient was subsequently lost to follow-up. Every patient demonstrated a clinical response at both time points. At 24 weeks, 50% achieved CR, with 41.7% of relapsing patients and 75% of newly diagnosed patients achieving this outcome. Nearly 70% attained a BVAS of 0. By week 76, the CR rate had risen to 81.3%, with all patients receiving ≤5 mg/day of oral prednisone and one patient entirely glucocorticoid-free. One relapse occurred at week 36. Detailed treatment regimens are provided in **Supplementary Table 1**.

**Table 2:** Primary outcomes for AAV patients who received obinutuzumab during follow-up.

Time	At week 24			At week 76		
Primary outcomes	Overall (N = 16)	Relapsing (n=12)	Newly diagnosed (n=4)	Overall (N = 16)	Relapsing (n=12)	Newly diagnosed (n=4)
CR rate, n (%)	8 (50)	5 (41.7)	3 (75)	13 (81.3)	10 (83.3)	3 (75)
Prednisone ≤7.5 mg/day	6 (37.5)	4 (33.3)	2 (50)	0	0	0
Prednisone ≤5 mg/day	4 (25)	2 (16.7)	2 (50)	13 (81.3)	10 (83.3)	3 (75)
No prednisone	0	0	0	1 (7.7)	1 (8.3)	0
Response rate, n (%)	16 (100)	12 (100)	4 (100)	16 (100)	12 (100)	4 (100)
Relapse rate, n (%)	0	0	0	1 (6.3)	1 (8.3)	0
BVAS						
BVAS = 0	11 (68.8)	8 (66.7)	3 (75)	13 (81.3)	10 (83.3)	3 (75)
BVAS = 2	2 (12.5)	2 (16.7)	0	0	0	0
BVAS = 4	3 (18.8)	2 (16.7)	1 (25)	2 (12.5)	1 (8.3)	1 (25)

AAV, anti-neutrophil cytoplasmic antibody (ANCA)-associated vasculitis; CR, complete remission; and BVAS, Birmingham Vasculitis Activity Score.

#### Secondary outcomes

**Figure 1:**
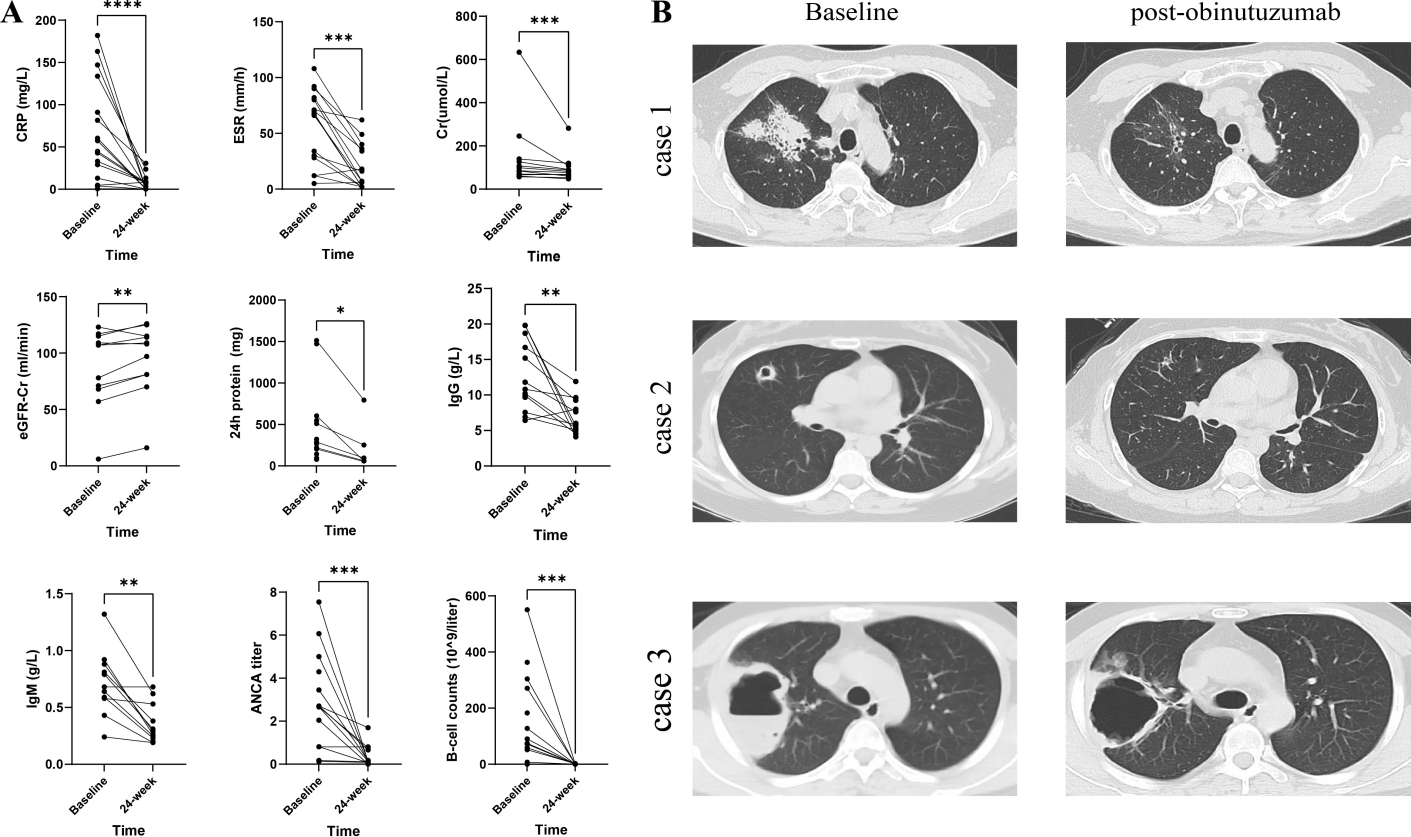
Laboratory parameters and chest CT images at baseline and 24 weeks post-obinutuzumab treatment. **(A)** Changes in the laboratory parameters of 16 patients at baseline and 24 weeks after obinutuzumab treatment. **(B)** High resolution CT (HRCT) images at baseline and post-obinutuzumab treatment (4, 8, and 12 weeks) in three cases. CRP, C-reactive protein; ESR, erythrocyte sedimentation rate; Cr, creatinine; ANCA, antineutrophil cytoplasm antibodies; eGFR, estimated glomerular filtration rate; IgG, immunoglobulin G; and IgM, immunoglobulin M. *p<0.05 **p<0.01, ***p<0.001 ****p<0.0001.

Secondary outcomes confirmed marked improvements in both systemic inflammation and immune parameters (**Figure 1A** and **Table 3**). Significant reductions in CRP and ESR levels were observed. Renal function, as measured by serum creatinine and eGFR, also notably improved. Although baseline proteinuria was only modestly elevated in our cohort, 24-h urinary protein excretion improved markedly after treatment. Additionally, improvement in lung condition was also observed (**Figure 1B**). Immunological analyses revealed profound B-cell depletion (median 74.9 × 10^9^/L pre-infusion versus 0 post-infusion; p = 0.0002) accompanied by a parallel decline in total immunoglobulin levels. The median immunoglobulin G (IgG) level decreased from 10.8 g/L (IQR 9.67–16.7) to 5.81 g/L (IQR 5.09–7.99; p = 0.0015), and all patients became ANCA-negative within 24 weeks of the first infusion.

**Table 3:** Comparative analysis of laboratory parameters within 24 weeks.

Variables, median (IQR)	Baseline	24 weeks	P value
CRP, mg/L	51.13 (25.13–101.77)	4.75 (0.28–8.47)	<0.0001
ESR, mm/h	67.50 (29.50–80.50)	11.50 (2–34.50)	0.0002
Serum Cr, µmol/L	92 (68.93–129.73)	79.50 (64.60–95.43)	0.0010
eGFR, mL/min	107 (69.50–112)	108 (81–114.50)	0.0070
24-h protein, mg	277.47 (157.76–538.05)	62.54 (57.14–174.69)	0.0313
IgG, g/L	10.80 (9.67–16.70)	5.81 (5.09–7.99)	0.0015
IgM, g/L	0.74 (0.59–0.83)	0.31 (0.26–0.58)	0.0020
ANCA, AU	2.67 (0.81–3.88)	0.13 (0.09–0.54)^#^	0.0005
B-cell counts, 10^9^/L	74.90 (55.30–226.60)	0 (0–0)	0.0002

IQR, interquartile range; CRP, C-reactive protein; ESR, erythrocyte sedimentation rate; Cr, creatinine; eGFR, estimated glomerular filtration rate; IgG, immunoglobulin G; IgM, immunoglobulin M; and ANCA, anti-neutrophil cytoplasm antibodies. ^#^The cutoff value for a positive ANCA titer was set at ≥1.

**Figure 2:**
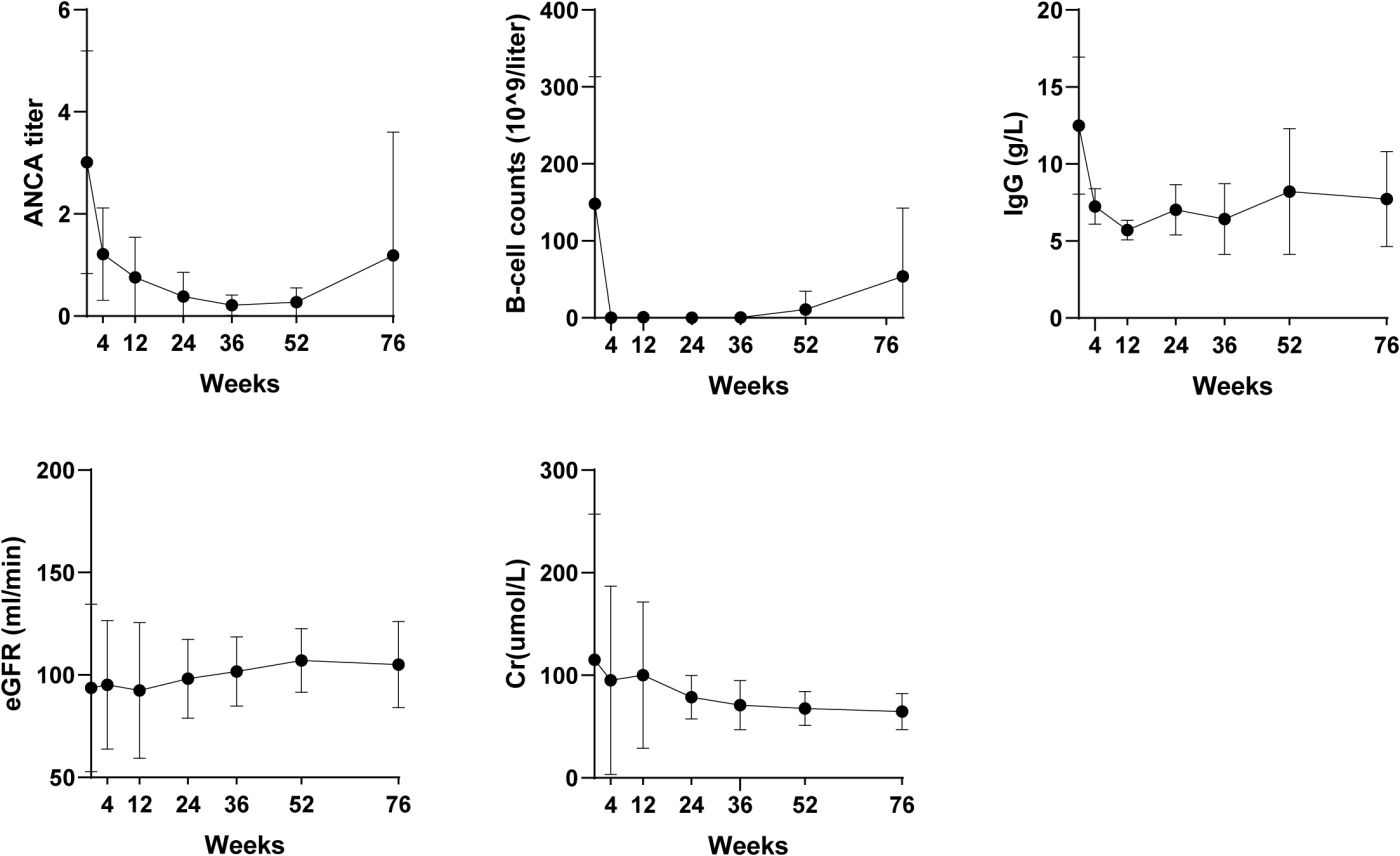
Temporal changes in five key laboratory parameters over 76 weeks. ANCA, antineutrophil cytoplasm antibodies; IgG, immunoglobulin G; eGFR, estimated glomerular filtration rate; Cr, creatinine.

Longitudinal analysis of five key biomarkers (ANCA titers, B-cell counts, IgG levels, eGFR, and serum creatinine) demonstrated durable therapeutic effects through 76 weeks (**Figure 2**). Rapid clinical improvement was evident within the first 4 weeks of obinutuzumab and persisted for approximately 52 weeks, as reflected by sustained ANCA negativity and profound B-cell depletion. Beyond week 52, a gradual rise in ANCA titers and B-cell repopulation was observed, indicating a potential return to baseline immune status.

### Adverse events

All patients received sulfamethoxazole–trimethoprim prophylaxis against *Pneumocystis jirovecii* pneumonia (PJP) beginning immediately after the first obinutuzumab infusion. No severe infections occurred. Infections occurred in seven patients (43.8%) with a median onset of 12 weeks (range: 5–25 weeks) after infusion. A total of 12 infectious episodes were recorded; four patients experienced recurrent infections. Viral infections predominated (7, 58.3%), followed by fungal (3, 25%) and bacterial (2, 16.7%) infections. Among the viral infections, three were caused by COVID - 19, two by cytomegalovirus (CMV), one by influenza A virus, and one by Epstein–Barr virus (EBV). The respiratory tract was the most common site of infection (9/12, 75%), with COVID - 19 accounting for half of these cases (**Table 4**).

**Table 4:** Infections documented during follow-up.

Infection	n, (%)
Patients who were infected	7 (43.8)
Infection times	12
Type	
Virus	7 (58.3)
COVID-19	3 (25)
CMV	2 (16.7)
Influenza A	1 (8.3)
EBV	1 (8.3)
Fungus	3 (25)
Bacterium	2 (16.7)
Site	
Respiratory tract	9 (75)
Urinary tract	1 (8.3)
Blood	2 (16.7)

CMV, cytomegalovirus, and EBV, Epstein–Barr virus.

A subgroup analysis examined potential risk factors for infection, including immunoglobulin levels, concurrent prednisone doses, and prior pulse methylprednisolone therapy (**Table 5**). Infections occurred predominantly during the induction phase and were associated with significantly higher daily prednisone doses (infected group: mean 55 mg/day vs. non-infected group: mean 18.3 mg/day; p = 0.0250), indicating that elevated glucocorticoid exposure increases the risk of infection. However, neither prior pulse methylprednisolone treatment nor immunoglobulin levels at the time of infection differed between the two groups.

**Table 5:** Risk factor analysis between the infected and non-infected groups.

Variables, mean (SD)	Infected (n=7)	Non-infected (n=9)	P value
IgG, g/L	6.2 (2.8)	7.4 (1.3)	0.3371
Concurrent prednisone, mg/day	55 (34.1)	18.3 (7.5)	0.0250
Pulse methylprednisolone, n (%)	5 (71.4)	4 (44.4)	0.3575

IgG, immunoglobulin G, and SD, standard difference.

Importantly, no patient developed severe hypogammaglobulinemia (IgG <4 g/L) during follow-up; nevertheless, five patients with a marked IgG decline (mean 5.3 g/L) received intravenous immunoglobulin supplementation. Three infusion-related reactions, namely, sinus bradycardia, transient hypertension, and a mild febrile episode, resolved promptly after reducing the infusion rate.

## Discussion

Obinutuzumab has already shown benefits for a spectrum of connective-tissue diseases, including systemic lupus erythematosus, anti-Jo1 syndrome, and *Calcinosis cutis*–Raynaud’s phenomenon–esophageal dysmotility–sclerodactylia–teleangiectasia (CREST) syndrome (17). In the present cohort, we extended these observations to refractory or relapsing AAV. At 76 weeks, CR was attained by 81% of the patients while successfully tapering prednisone to ≤5 mg/day. This was accompanied by marked reductions in ANCA titers and preservation of renal function. Our results are consistent with a case series in which three rituximab-refractory AAV patients achieved sustained remission after treatment with obinutuzumab (18).

Elevated glucocorticoid exposure is a well-established risk factor for infections. Guided by this principle, we adopted an aggressive tapering strategy: oral prednisone was initiated at 1 mg/kg/day (maximum 60 mg) and reduced to 30 mg/day by week 4 and approximately 15 mg/day by month 3, whenever clinically feasible. In this real-world cohort, the precise schedule remained at the treating physician’s discretion. Consequently, attainment of CR (prednisone ≤ 10 mg/day) often took longer than in controlled trials. Consistent with prior reports, infections clustered during the induction period and correlated with significantly higher daily prednisone doses.

Long-term follow-up results revealed that, although ANCA titers and B-cell counts began to rebound after 52 weeks, both Cr and eGFR levels remained stable through 76 weeks. This stability further supports the potential role of obinutuzumab in preserving kidney function. This benefit is especially relevant for the MPA subset, which carries the highest renal risk and is disproportionately prevalent in Asia. In our cohort, all four MPO-ANCA-positive patients, each of whom was previously refractory to CTX or RTX, achieved CR by week 76 while maintained on only 5 mg/day prednisone, highlighting obinutuzumab’s superior efficacy in treating this refractory population.

To minimize the risks of adaptive immune dysfunction and susceptibility to opportunistic infections, we administered prophylactic medications, such as sulfamethoxazole-trimethoprim prophylaxis for PJP prevention, isoniazid for tuberculosis prevention, and entecavir for hepatitis B prevention. Additionally, we would recommend vaccination for every patient, although it is not mandatory. Despite pre-medication with intravenous methylprednisolone and paracetamol to mitigate severe infusion reactions, three infusion-related adverse events were documented. All were mild, and each resolved after a transient reduction in infusion rate. Obinutuzumab-induced thrombocytopenia (19) has been well-documented in a previous study. However, this symptom was not observed in our cohort.

A key strength of this study is that it represents the largest real-world cohort of refractory or relapsing AAV patients treated with obinutuzumab to date. Despite this strength, the small sample size constrained the statistical power and limited the generalizability of our findings. Furthermore, the reduced-dose obinutuzumab regimen employed was deliberately more conservative compared with lymphoma protocols, reflecting its status as an exploratory, non-standard approach for AAV. While this lower-dose strategy may potentially reduce adverse effects, it also raises concerns regarding its ability to achieve optimal therapeutic efficacy. The absence of an evidence-based dosing framework for obinutuzumab in AAV underscores the urgent need for well-designed, randomized, controlled trials to establish standardized, effective, and safe treatment protocols. Nonetheless, this exploratory approach represents an important step toward understanding the potential role of obinutuzumab in managing refractory AAV. In our study, CR was achieved when a patient was maintained on a prednisone dosage of ≤10 mg/day. However, more stringent criteria are now favored in current clinical research and practice. The 2022 EULAR update recommends that patients with AAV achieve a dosage of ≤5 mg/day by 4–5 months (3). Furthermore, the TAPIR trial (20) supports the notion that a dosage of ≤5 mg/day, or ideally complete glucocorticoid discontinuation, may represent a more contemporary and clinically relevant definition of remission. Nevertheless, this study offers critical real-world evidence supporting obinutuzumab as an experimental treatment option for AAV, leveraging its potent B-cell-depleting activity. Future investigations should prioritize dose optimization strategies, rigorous immune function surveillance, and extended follow-up to definitively establish the therapeutic role of obinutuzumab in AAV.

## Conclusions

Reduced-dose obinutuzumab demonstrated rapid and durable efficacy with a favorable safety profile in patients with refractory or relapsing active AAV, establishing it as a promising therapeutic alternative, particularly after RTX or CTX failure.

## Data Availability

The data supporting the findings of this study are available within the article, further inquiries can be directed to the corresponding author.
