# Supplementary material for: Reduced-dose obinutuzumab induces remission in refractory ANCA-associated vasculitis: a report of 16 cases
Source: Front Immunol. 2025 Aug 29;16:1624234. doi: 10.3389/fimmu.2025.1624234 (PMC12426241; doi:10.3389/fimmu.2025.1624234)
Supplement: Supplementary file 1 [file Table1.docx]

**Supplementary Table 1. Detailed treatment regimens of 16 patients with ANCA-associated vasculitis treated with obinutuzumab.**

|  | Gender | Age | ANCA | BVAS | Remission inducing treatment | Obinutuzumab administration | Maintenance treatment | 24-week | | 76-week | |
| --- | --- | --- | --- | --- | --- | --- | --- | --- | --- | --- | --- |
|  |  |  |  |  |  |  |  | CR | pred | CR | pred |
| Case 1 | Male | 31 | PR3 | 7 | CTX,MMF,RTX | 1000mg |  | No | 20 | No | 15 |
| Case 2 | Male | 30 | PR3 | 26 | CTX,MMF | 1000mg | RTX | No | 15 | Yes | 5 |
| Case 3 | Male | 29 | PR3 | 12 | MTX | 1000mg | RTX | No | 17.5 | Yes | 5 |
| Case 4 | Male | 57 | MPO | 15 | AZA,CTX | 2000mg, 2 weeks |  | No | 15 | Yes | 5 |
| Case 5 | Female | 22 | PR3 | 16 | AZA,CTX | 1000mg |  | No | 15 | Yes | 0.8 |
| Case 6 | Male | 61 | PR3 | 18 | MMF,CTX | 1000mg | RTX | Yes | 7.5 | Yes | 2.5 |
| Case 7 | Male | 33 | MPO | 22 | MMF,RTX,CTX,CsA | 2500mg, 40 weeks |  | Yes | 10 | Lost |  |
| Case 8 | Female | 46 | PR3 | 9 | RTX,MTX,AZA | 1000mg |  | Yes | 10 | Yes | 0 |
| Case 9 | Female | 48 | MPO | 13 | MMF,CTX | 1000mg |  | Yes | 10 | Yes | 5 |
| Case 10 | Male | 68 | MPO | 18 | LEF,MTX,CTX,AZA,RTX | 1000mg | Tocilizumab | No | 20 | Yes | 5 |
| Case 11 | Male | 43 | PR3 | 22 | MTX,CTX | 2000mg, 76 weeks |  | Yes | 7.5 | Yes | 4 |
| Case 12 | Female | 37 | PR3 | 13 |  | 2000mg, 76weeks |  | Yes | 10 | Yes | 5 |
| Case 13 | Female | 31 | PR3 | 10 | CTX | 1000mg |  | Yes | 5 | Yes | 5 |
| Case 14 | Male | 54 | PR3 | 24 |  | 1000mg |  | No | 20 | No | 15 |
| Case 15 | Female | 49 | PR3 | 20 |  | 1000mg |  | Yes | 10 | Yes | 5 |
| Case 16 | Male | 55 | PR3 | 20 |  | 1000mg |  | Yes | 5 | Yes | 5 |

ANCA, anti-neutrophil cytoplasmic antibody; BVAS, Birmingham Vasculitis Activity Score; PR3, proteinase 3; MPO, myeloperoxidase; CTX, cyclophosphamide; MMF, mycophenolate mofetil; RTX, rituximab; MTX, methotrexate; AZA, azathioprine; CsA, cyclosporin A; LEF, leflunomide; CR, complete remission; pred, prednisone
